# Non-Deterministic Assessment of Surface Roughness as Bond Strength Parameters between Concrete Layers Cast at Different Ages

**DOI:** 10.3390/ma13112542

**Published:** 2020-06-03

**Authors:** Janusz Kozubal, Roman Wróblewski, Zbigniew Muszyński, Marek Wyjadłowski, Joanna Stróżyk

**Affiliations:** 1 Faculty of Civil Engineering, Wroclaw University of Science and Technology, 50-370 Wrocław, Poland; janusz.kozubal@pwr.edu.pl (J.K.); marek.wyjadlowski@pwr.edu.pl (M.W.); joanna.strozyk@pwr.edu.pl (J.S.); 2 Faculty of Geoengineering, Mining and Geology, Wroclaw University of Science and Technology, 50-370 Wrocław, Poland; zbigniew.muszynski@pwr.edu.pl

**Keywords:** concrete, roughness, texture, close-range photogrammetry, bond strength, random field generation, semivariograms

## Abstract

The importance of surface roughness and its non-destructive examination has often been emphasised in structural rehabilitation. The presented innovative procedure enables the estimation of concrete-to-concrete strength based on a combination of low-cost, area-limited tests and geostatistical methods. The new method removes the shortcomings of the existing one, i.e., it is neither qualitative nor subjective. The interface strength factors, cohesion and friction, can be estimated accurately based on the collected data on a surface texture. The data acquisition needed to create digital models of the concrete surface can be performed by terrestrial close-range photogrammetry or other methods. In the presented procedure, limitations to the availability of concrete surfaces are overcome by the generation of subsequential Gaussian random fields (via height profiles) based on the semivariograms fitted to the digital surface models. In this way, the randomness of the surface texture is reproduced. The selected roughness parameters, such as mean valley depth and, most importantly, the geostatistical semivariogram parameter sill, were transformed into contact bond strength parameters based on the available strength tests. The proposed procedure estimates the interface bond strength based on the geostatistical methods applied to the numerical surface model and can be used in practical and theoretical applications.

## 1. Introduction

Concrete layers of considerably different ages are frequently used in new and existing (strengthening) structures. The predominant shear action appears in structural elements made from a combination of precast and cast in place concrete like, for example, floor systems, slab renovation or bridge decks. The same action and an even greater age difference appear in reinforced or repaired structures by increasing their thickness. The joints made during concreting are another example but neither the age of concrete layers at the interface is different nor is the shear dominant. Instead, tension is usually a major action.

There exist several reasons why research on the adhesive action of concrete is important:

- It requires standardisation due to its importance in practice.
- Aggressive contaminants may penetrate the delaminated interface and cause unfavourable conditions for durability.
- Element stiffness may be reduced and larger deformations appear, reducing serviceability.
- Load transfer between layers changes with the bond failure.
- Shrinkage is a factor that influences pure cohesive action and element deformations.

Concrete structures consisting of layers cast at various ages require a different approach than other concrete structures in design and construction. This approach includes contact surface preparation, construction stages, curing, load resistance, etc., and the most important factor is the bond strength of two concrete layers because it is critical for the durability and load-bearing capacity. Besides its material strength, the way the surface of the existing concrete is prepared has a significant influence on its element capacity and overall durability due to the random distribution of material and interface parameters over the contact surface.

Since the bond is a relatively low action, it is also sensitive to several factors, like roughness and tortuosity in the contact area, concrete composition, density, strength, porosity and micro-damages of the interfacial zone. Among those factors, surface roughness and its condition determine concrete-to-concrete interface behaviour. Being essential, substrate surface condition assessment is the subject of extensive research focused on friction and cohesive actions. Frictional action strongly depends on the interlock of aggregate particles [1] and thus is mostly activated after delamination. At the same time, existing reinforcement is activated and dowel action with compression that is normal to the interface appears. The cohesive action, originated by chemical and other connections, bonds the surfaces before their delamination and enables element continuity. With this in mind, the methods used to obtain internal forces in ultimate and serviceability limit states should be different. Linear elastic models are better suited to serviceability limit states with continuous stress distribution at the interface. However, this assumption is no longer valid in the ultimate limit state when delamination occurs.

According to the shear friction theory proposed in [2] and successfully used in design codes, the transfer mechanism at the concrete-to-concrete interface is ensured by friction and is a subject of simultaneous shear and compression forces as in [3], where the load transfer mechanism of shear forces consists of:

- Cohesion, due to mechanical interlocking between particles;
- Friction, due to the existence of compression stresses at the interface and due to the relative displacement between concrete parts;
- Dowel action, due to the deformation of the reinforcement bars crossing the interface.

Thus, the ultimate limit state shear stress at the interface $v_{Rdi}$ is represented by the following equation:

(1) $$v_{Rdi}=c\cdot f_{ctd}+\mu \cdot \sigma_{n}+\rho \cdot f_{yd}\cdot \left(\mu \cdot \sin\alpha +\cos\alpha \right)$$

where $f_{ctd}$ is the design tensile strength of concrete (and is not specified if in precast or cast in place), $\sigma_{n}$ is the external normal stress acting on the interface, ρ is the reinforcement ratio, $f_{yd}$ is the yield strength of the reinforcement and α is the angle between the shear reinforcement and the shear plane. The other symbols, *c*—cohesion factor and *μ*—friction factor, are the factors that depend on the roughness of the interface and are classified according to three categories based on the surface finishing. The surface categories are, to some extent, arbitrary and can lead to an inaccurate assessment of cohesion and friction (usually too conservative). Moreover, it is not clear if the cohesion and friction are average or design values, so they are not characterized statistically. Taken together, the evaluation of the factors is only qualitative.

Model Code 2010 [4] improves surface qualification based on the average roughness *R_a_* where a smooth surface is defined with *R_a_* < 1.5 mm, rough with *R_a_* ≥ 1.5 mm and very rough/indented with *R_a_* ≥ 3 mm. A very smooth surface is not defined. However, as noted in [5], the average roughness *R_a_* is not sensitive enough. It does not provide information on the local variability and different profiles can present the same *R_a_*.

One of the first quantitative approaches is presented in [6,7]. In [6], the contact stress depends on the matrix proportions of maximum particle size and volumetric percentage of aggregate. Moreover, the contact areas are obtained from the probability density functions of the particles’ (aggregate) occurrence in the surface plane, thus the method has a probabilistic approach. The model presented in [7] is based on concrete strength, crack width and maximum aggregate size. Therefore, both methods require the assessment of surface parameters.

Since shear friction theory is well established, recent research [8,9] has been focusing on the evaluation of the design coefficients to overcome qualitative assessment. These limitations have been overcome by the calculation of the *c* and *μ* factors based on surface roughness parameters such as the mean valley depth *R_vm_.* Moreover, the design values of both the *c* and *μ* factors have been provided in [10]. The design parameters have also been derived for a trilinear model which is proposed in [11]. The model is based on a parametric analysis of the available tests. The design coefficients for monotonic and cyclic loading have been calibrated to obtain the target values of the reliability index. The proposed model is also consistent with [12].

Further improvement in surface evaluation methods would include joint micro- (roughness from 1 μm to 0.5 mm) and macro-texture (waviness from 0.5 mm to 50 mm) analysis. Waviness and roughness can be related to different frequencies and wavelengths. The accuracy of the close-range photogrammetry used in this paper is on the border of these intervals, with a predominance of waviness, but other methods are also available [13,14]. Contact, X-ray and optical methods like profilometry, photogrammetry and scanning can be used to measure the surface parameters through point, linear or surface measurements. In this paper, the 3D surface parameters are used to evaluate the shear strength parameters at the interface of the concrete layers.

Surface scanning in laboratory conditions, as presented in [8], can be a preliminary step before other tests; for example, pull-off, as presented in [15,16,17]. Destructive tests, such as tension, shear and a combination of shear and compression, are also possible [10]. Part of the extensive research described in [5] was performed with a profilometer and roughness parameters were obtained from the 2D analysis. The samples were then subjected to destructive tests to determine the shear strength. Maximum valley depth *R_v_* was found to be the most adequate surface parameter and presented an almost linear correlation with the concrete bond strength in shear. However, in order to avoid problems with possible strong surface irregularities, mean valley depth *R_vm_* was preferred.

One of the advantages of non-destructive methods is the reliable determination of waviness and roughness parameters from relatively small sample sizes. These measurement techniques are accurate and time-saving, but they may also be time-consuming when they are numerically post-processed. However, the possible evaluation of strength parameters based on the concrete surface examination is very promising. Moreover, it is possible to carry out the tests in situ or in laboratory conditions, and the in situ examination can be used as a quality check. This problem is also essential in structural rehabilitation and strengthening with composite materials [18].

The proposed roughness assessment was sought independently of the chosen measurement method. Attempts were made to eliminate subjective elements from the measurement and compose a flexible set of procedures. The whole method is compatible with surface monitoring tools used in civil engineering, such as 3D laser scanners and confocal microscopes, and resistant to possible human error.

Although there are extensive test data available, theoretical results do not follow the same degree of accuracy. Therefore, we established a method combining geostatistical image analysis and aggregate composition with close-range photogrammetry (CRP) and leading to the non-destructive method of bond strength assessment. Therefore, this paper aims to improve existing non-destructive methods of concrete surface examination with:

- More detailed investigation—both roughness and waviness are considered, so profile height and inclination variability are identified.
- Application of random surfaces, i.e., storing information on surface texture in a random image of a real surface.
- Introduction of statistical objective parameters instead of traditional roughness parameters in order to reproduce the randomness of any surface texture.

In the presented method, the experimental research and sampling may be limited due to the application of geostatistics which predicts data values at unsampled locations. Furthermore, the geostatistical image of a real surface is used to obtain a required texture/roughness parameter. Then, interface strength parameters are derived based on the available test data and the texture/roughness parameter.

The geostatistical methods [19,20,21] involved in predicting surface images are gaining importance in existing structures when examined surfaces cannot be easily accessed or used as a quality check. Moreover, apart from practical applications, it is possible to use the generated images in reliability problems, so we present the developed source code in the R language [22] to allow application of the presented algorithms.

## 2. Materials and Methods

### 2.1. Concrete Mix Composition

**Figure 1:**
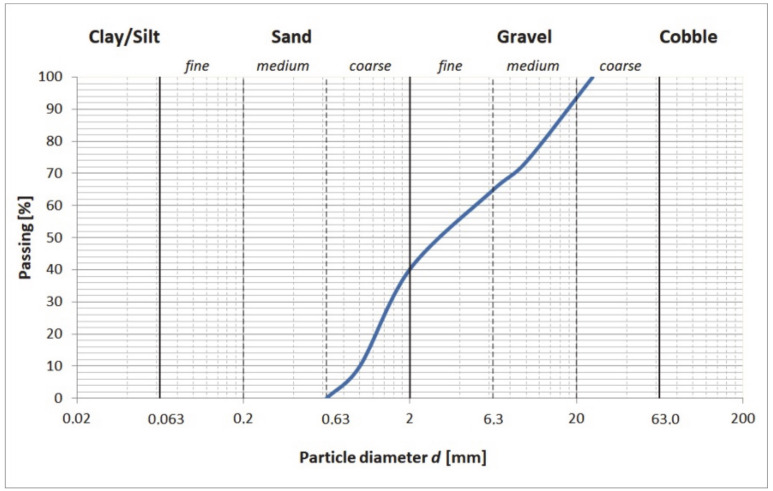
Particle size distribution of aggregates according to sieve analysis.

Concrete samples with the composition and density presented in Table 1 were used in the research. The cement used for the concrete mix was the CEM I 42.5R (Górażdże Cement S.A., Górażdże, Poland) with a specific surface area of 375.2 m^2^/kg and the following compressive strength: 2-days 26.6 MPa and 28-days 56.0 MPa. Moreover, the cement consisted of 2.83% of sulfur trioxide, 0.044% of chloride ion, 0.61% of total alkali and 2.11% of ignition loss. The aggregate was composed of fine and coarse particles. The particle size content curve obtained from the sieve analysis is presented in Figure 1. While the fine aggregate consisted mostly of sand with a grain size of 1/2 mm, the sand constituted 40.2% of the aggregate by weight. Furthermore, the fine aggregate particles were natural sand and were usually rounded and well-rounded in the cubic or occasionally elongated form [23], and the surface texture of these particles was smooth. Sand grains were mainly individual minerals such as quartz and feldspar.

**Figure 2:**
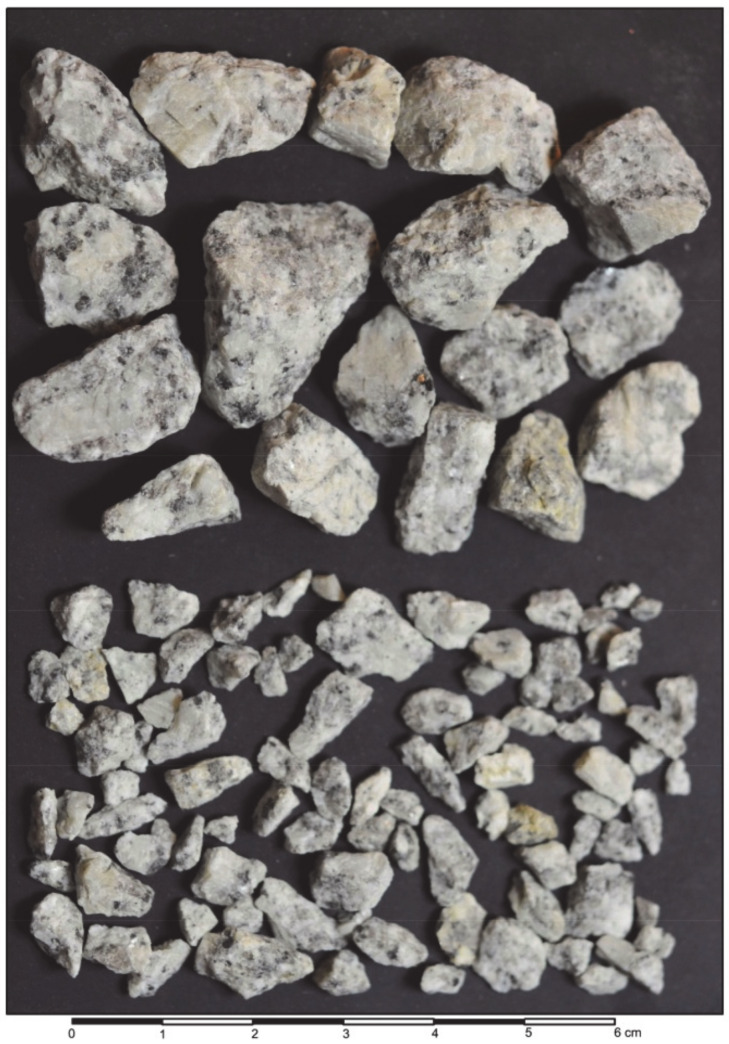
Granite fragments of coarse aggregates: medium and fine gravel.

The remaining 59.8% was the coarse aggregate of crushed granite in two fractions: fine gravel (24.6%) and medium gravel (30.2%), as presented in Figure 2. These particles were angular and very angular in elongated and slightly flat forms with rough surface textures. Their shape index SI according to [24] was low, e.g., for medium gravel, SI = 16.

### 2.2. Samples

**Figure 3:**
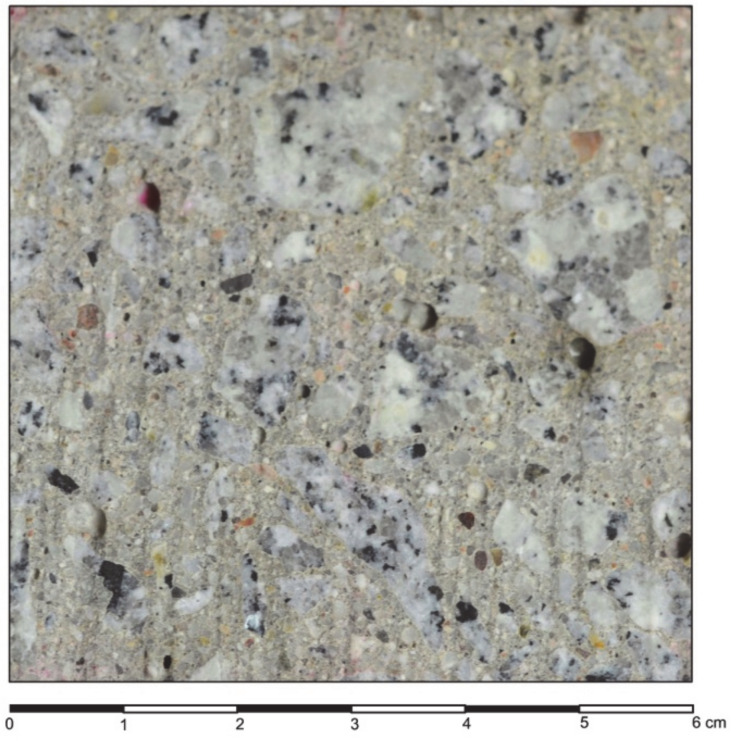
A 60 × 60 mm^2^ fragment of the water cut concrete sample surface.

**Figure 4:**
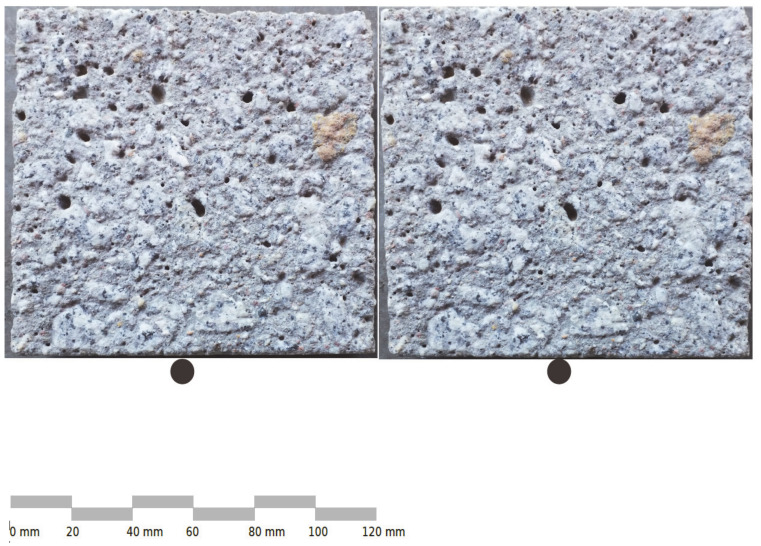
Stereoscopic image of the sandblasted surface (together, the left and right images create a 3D effect while keeping eyes as “looking into the distance”).

Cube concrete samples of 150 × 150 × 150 mm^3^ were prepared. They matured in water for 28 days and after another 60 days, smaller samples were water cut (Figure 3) to a size of 120 × 120 × 30 mm^3^. The cut sample surfaces of 120 × 120 mm^2^ were further processed. Sandblasting was chosen from many available surface treatment methods. This method is a popular process for concrete processing in technical applications and it transforms the surface randomly in an isotropic way. Sandblasting was performed using a standardized nozzle, pressure and aggregate grain size in order to repeat the experiment in the future. During the sandblasting with sand grains with d = 0.10–2.00 mm and pressure 0.6 N/mm^2^, an attempt was made to maintain a uniform regime, i.e., constant distance of the nozzle from the surface and constant speed of its movement to obtain even surfaces, as presented in Figure 4.

**Figure 5:**
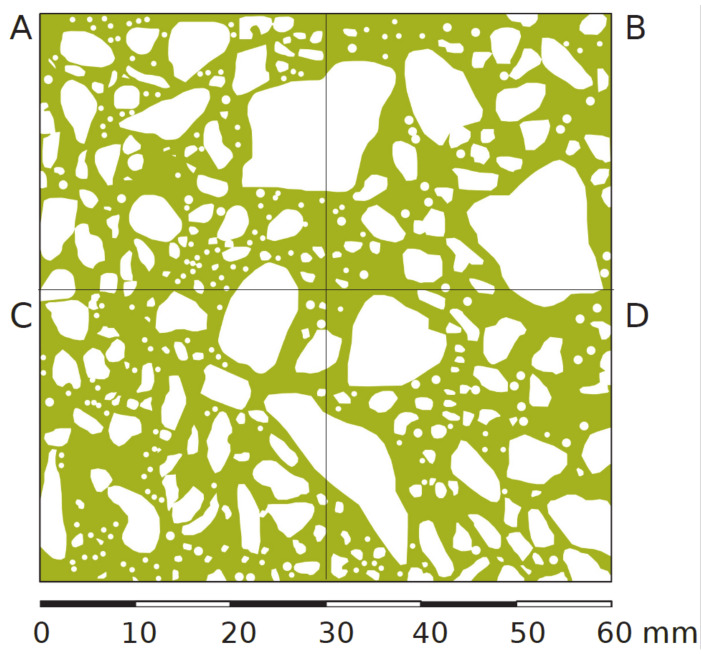
The 2D model of the concrete surface presented in Figure 3 (green—acement matrix, white—aggregates). (**A**–**D**): four segments of the sample.

The surface area ratios of the concrete components of the aggregate and cement matrix were estimated on the sand processed surfaces with 2D image analysis. The aggregate and the cement matrix ratios were assessed, as presented in Figure 5 and Table 2, in areas A, B, C and D. The ratio of the aggregate surface varied from 41.81% to 49.48%.

Afterward, five surfaces of the five samples were used in the research. One of the surfaces, P0, was not processed (mold touching left) and the others (P20, P21, P22, P23) were sandblasted after water cutting.

### 2.3. Background of the Proposed Method

Based on samples of limited dimensions, the presented method detects surface parameters and extends the results to a larger concrete area. The developed approach includes a combination of the following methods:

- point cloud acquisition (with known coordinates (*x,y,z*) representing sample surface) with an adequate accuracy;
- surface assessment with use of geostatistical methods, i.e., fitting of theoretical semivariogram to empirical data;
- generation of Gaussian random field (via height profiles) based on the fitted semivariogram;
- computation of the roughness/texture parameters based on the generated profiles or surfaces;
- correlation analysis of the semivariogram parameters with shear strength factors throughout roughness/texture parameters.

**Figure 6:**
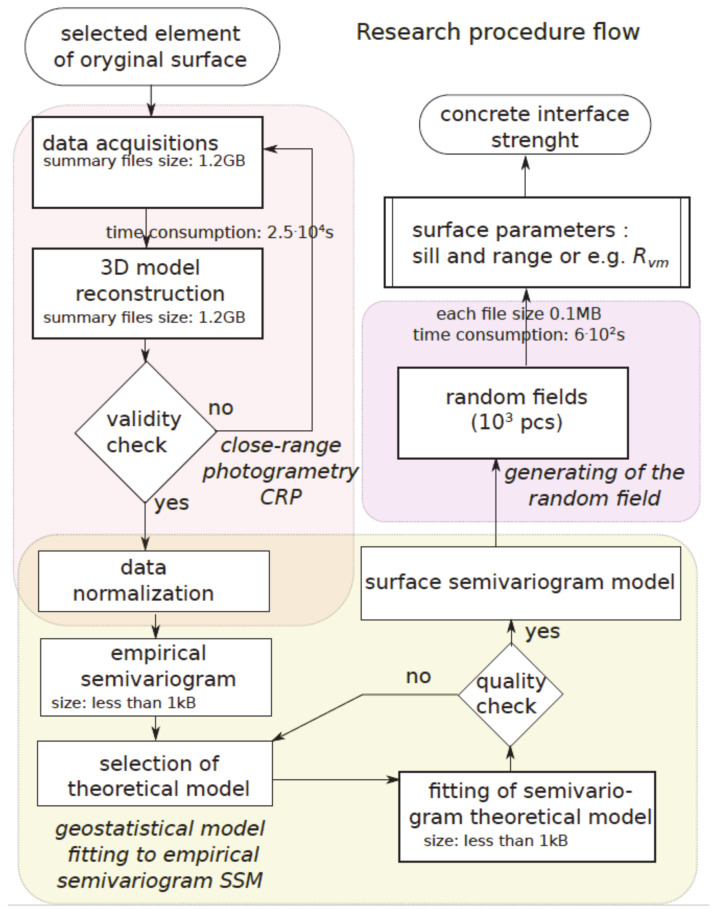
Block diagram of the shear strength parameter assessment.

In this paper, determination of the surface parameters, i.e., point cloud coordinates and profile height (*x,y,z*), was obtained using close-range photogrammetry. The entire procedure of the assessment of the concrete shear strength parameters is presented in Figure 6.

### 2.4. Data Acquisition Employing Photogrammetry

**Figure 7:**
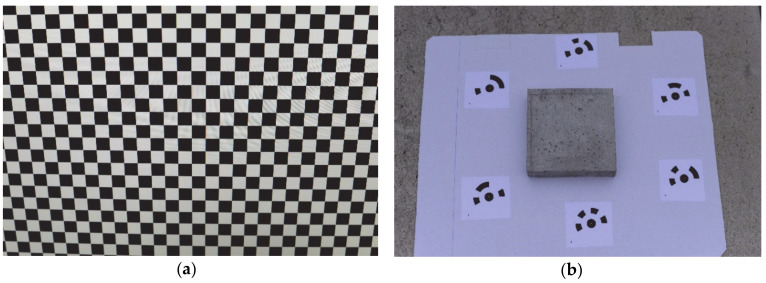

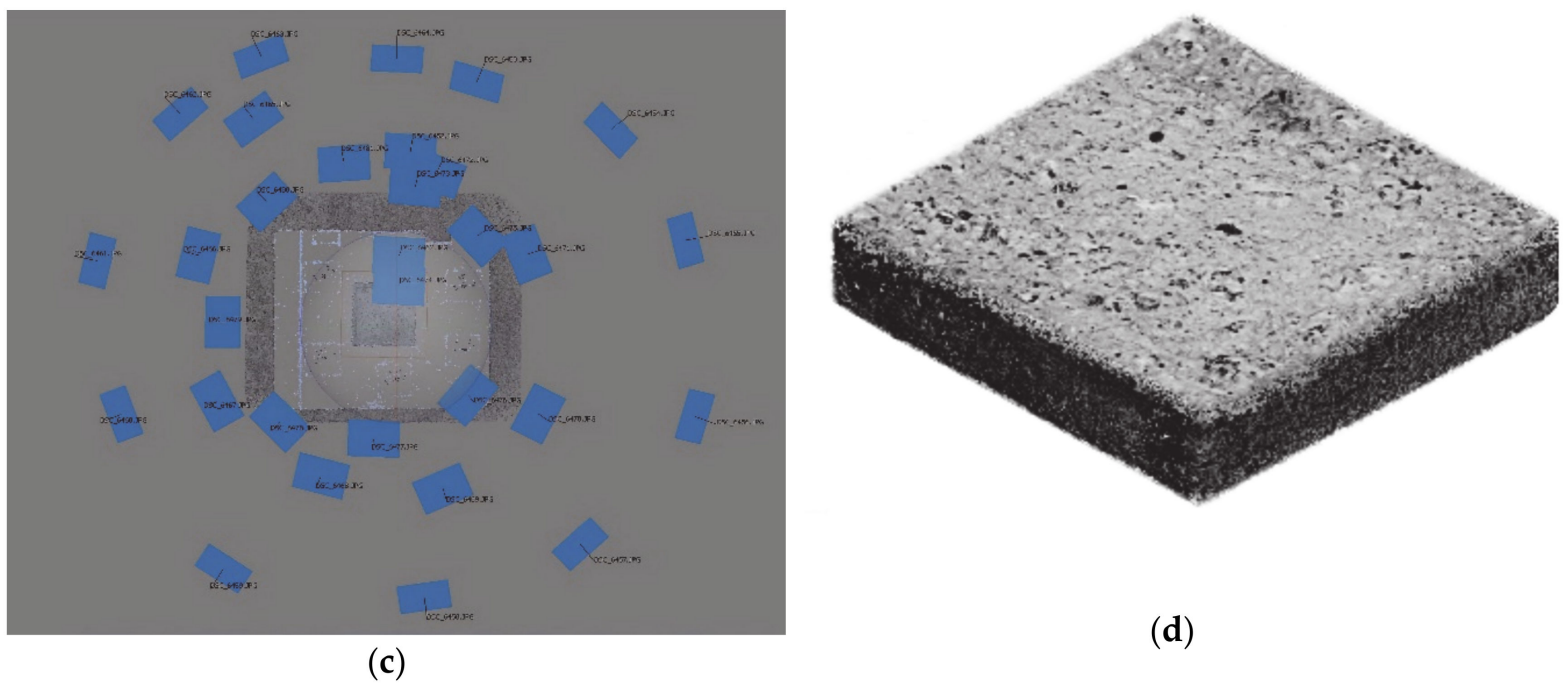
Stages of close-range photogrammetry (CRP) model preparation: (**a**) black and white chessboard for camera calibration; (**b**) a sample on the stand with markers (six coded control points); (**c**) estimated locations of the camera (blue rectangles); (**d**) dense point cloud obtained for the concrete sample.

Terrestrial close-range photogrammetry (CRP) is widely used in civil engineering. Professional photogrammetric cameras equipped with high-quality lenses take metric photos with known orientations in the adopted reference system. These photos include elements of internal orientation, which allows researchers to read the coordinates of the photographed objects. Based on these coordinates, it is possible to control the shape of structural elements and their location in space. Nowadays, another approach is emerging in photogrammetry which involves the use of the so-called non-metric cameras with cheaper lenses that are characterized by greater distortions (e.g., radial, tangential). Geometric distortions are minimized in the camera calibration process based on the analysis of many photos presenting special calibration charts (e.g., black and white chessboard), as presented in Figure 7a. The lack of elements of internal and mutual orientation is replaced by a large number of photos taken, which overlap to a large extent (covering almost the same area as the object). Automatic detection of the tie points on adjacent photos allows researchers to estimate the camera position and orientation in space. It allows them to build a 3D model of the measured object. To obtain the correct scale of the model, as well as to optimize the estimated camera position and provide appropriate georeferencing, the control points are established on the object. The known coordinates of the control points (usually determined using an electronic total station) also provide the possibility of checking the accuracy of the created 3D model of the object. The obtained model’s accuracy depends on many of the following factors: size of the object, shooting distance, camera matrix resolution and lens parameters, number of control points, their arrangement and coordinate accuracy, as well as the lighting, colour and texture of the photographed object.

In the literature, there are many examples of photogrammetry applications in construction and civil engineering. The usefulness of CRP in crack detection and the accurate 3D modelling of medieval bridge elements as well as for exterior material characterization were analysed in [25]. An example of a CRP application for the deformation analysis of a concrete beam during a loading experiment is presented in [26]. Precise estimation of the vertical deflections and horizontal displacements of the concrete beam during the load test in laboratory conditions showed that the photogrammetry technique was capable of monitoring both static and dynamic deformations [27]. Nowadays, photogrammetry is increasingly used for measurement of the vibrating structures. The most current trends in this technique are point tracking, digital image correlation and targetless approaches, as well as the integration of photogrammetry with other measurement techniques used in structural dynamics (e.g., interferometry) [28]. Both the point tracking technique and the development of a 3D model of the object based on a set of photos require an effective algorithm for recognizing the control point markers on the pictures. The aforementioned desire to integrate various measurement techniques usually involves combining photogrammetry with laser scanning. A coupled 3D laser scanning and digital image correlation system for the geometry acquisition of a railway tunnel was proposed and tested in [29]. When integrating measurement methods, attention should be paid to the accuracy achieved by the individual techniques and, in particular, this accuracy should be checked in real conditions prevailing at the construction site [30].

In the presented research, the photos of the concrete surface samples (120 × 120 mm^2^) were taken with a Nikon D800 non-metric camera (Nikon CEE GmbH, Warsaw, Poland) with a 50 mm single-focal-length lens with a resolution of 7360 × 4912 pixels. To calculate the pre-calibration parameters (in Agisoft Lens software, Version 0.4.2 beta 64 bit (build 2399), Agisoft LLC, St. Petersburg, Russia [31]), 10 photos of a black and white chessboard were taken (Figure 7a). Next, for each concrete sample, several dozen photos were taken using a special flat stand and six markers (control points) (Figure 7b). The coordinates of the control points in the local coordinate system were determined using the least square method (LSM) through the adjustment of 15 linear measurements. The horizontal position accuracy of the control points after adjustment was no greater than 1 mm. For each concrete sample, the tie points on the photos were found with the highest accuracy requirement and all photos were aligned in the Agisoft PhotoScan Professional software (Version 1.2.4 64 bit (build 2399), Agisoft LLC, St. Petersburg, Russia) [32]. Examples of the estimated positions of the camera when pictures of the concrete sample were taken are presented in Figure 7c as blue rectangles. The obtained tie points were filtered using the values of reprojection error, reconstruction uncertainty and projection accuracy. Based on control points, the optimization of camera alignment, as well as the scaling and georeferencing of the samples, were performed. The last step was to generate a dense point cloud for each concrete sample, an example of which is presented in Figure 7d.

The CSV format files were generated from the results of the photogrammetric measurement of the concrete samples. Each file contained a collection of points representing the sample surface (3D model) supplemented with colour information components in the form of records (*x*,*y*,*z*,R,G,B). The collected *z* coordinates of the points were normalized in reference to an average value of *z* or in reference to a surface trend if necessary.

### 2.5. Geostatistical Models of the Sample Surfaces

To fit the theoretical semivariograms to empirical data from 3D surface models, geostatistical methods were used. The semivariograms measured the spatial autocorrelation of the acquired sample points. The measured height, i.e., the “*z*” values, were used to build an empirical semivariogram with the LSM and the Gauss–Newton algorithm as a nonlinear fitting method. The general form of the semivariograms according to Equation (2) was used [33], where for each *h*, half of the mean value of the squared difference $\left(z\left(d\right)-z\left(d+h\right)\right)$ is defined as semivariance with a squared length unit.

(2) $$\gamma \left(h\right)=\frac{1}{2N}\overset{N}{\underset{i=1}{\sum }}{\left(z\left(d\right)-z\left(d+h\right)\right)}^{2}\;\;\;\left\{d,d+h\right\}\in P\;\;$$

where *z(d)* are normalized “z” coordinates at location *d, h* is the lag distance, i.e., distance that separates the two analysed locations and *N*—number of tested pairs {*d, d + h*} from space ***P***.

**Figure 8:**
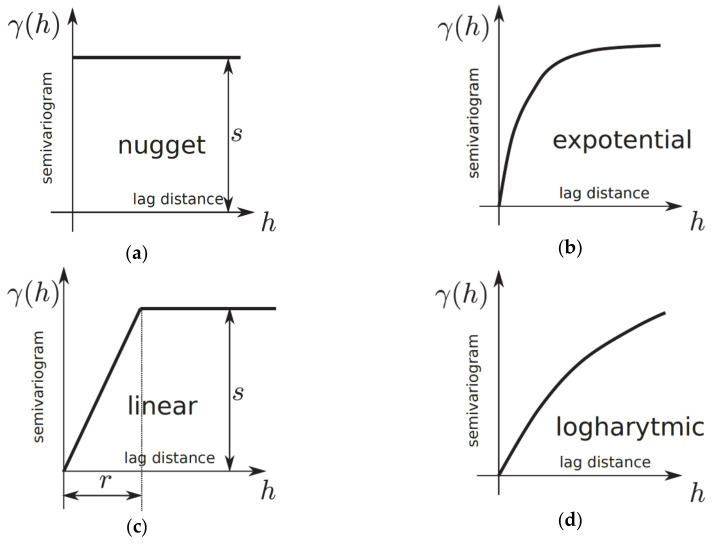

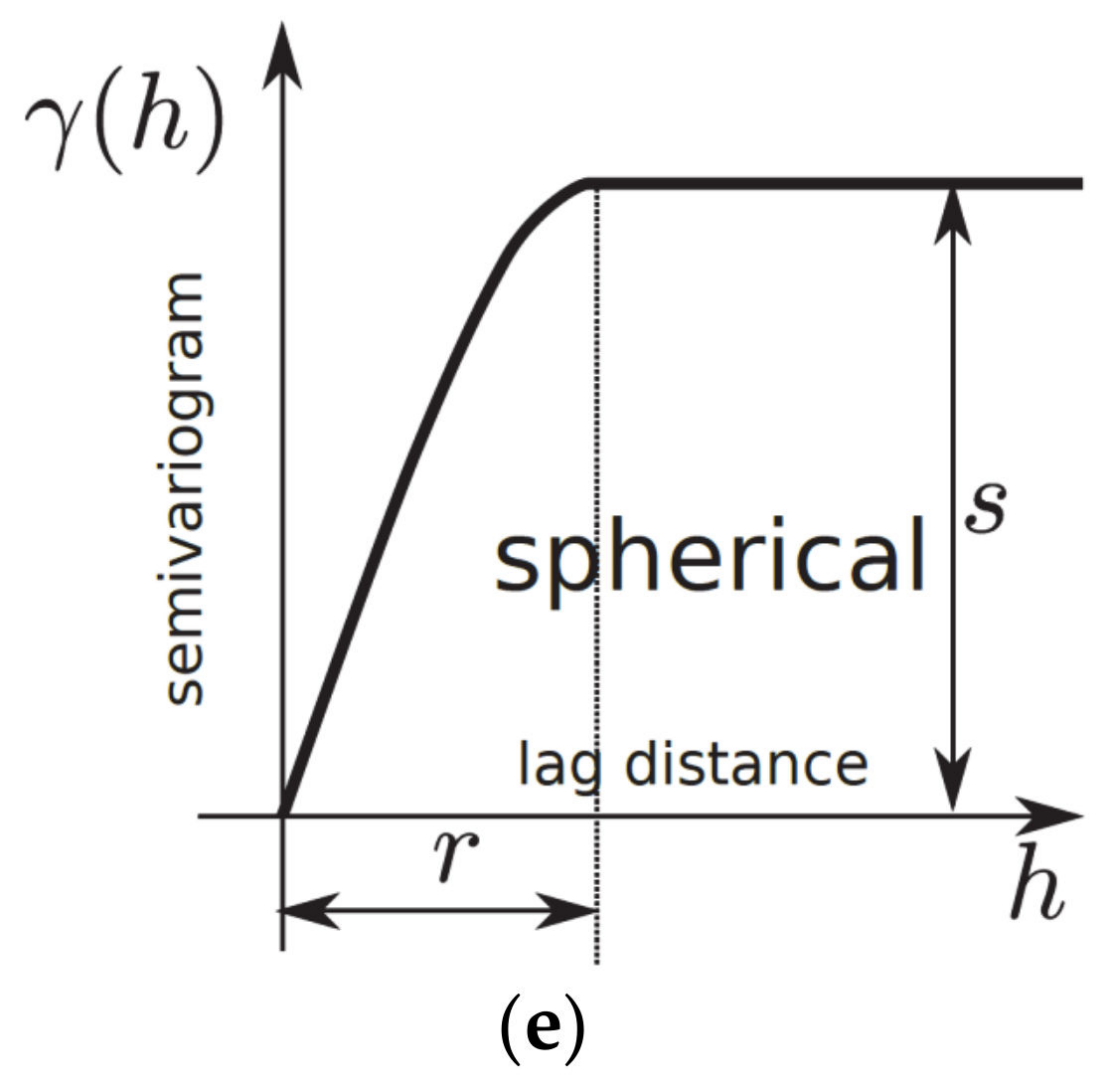
Theoretical semivariogram models (graphs): (**a**) nugget; (**b**) expotential; (**c**) linear; (**d**) logarithmic; (**e**) spherical.

For any two locations in a small distance on a surface, a small value of a measure ${\left(z\left(d\right)-z\left(d+h\right)\right)}^{2}$ is expected. With the increasing lag distance *h* between points, the similarity of the measure decreased. This natural behaviour could be described by various theoretical relationships, as presented in Table 3 and Figure 8. The nugget model is used for discontinuous characteristics or areas of limited local extend. Spherical, exponential, Gaussian and linear-plateau models are important among the commonly used semivariograms in engineering applications.

**Figure 9:**
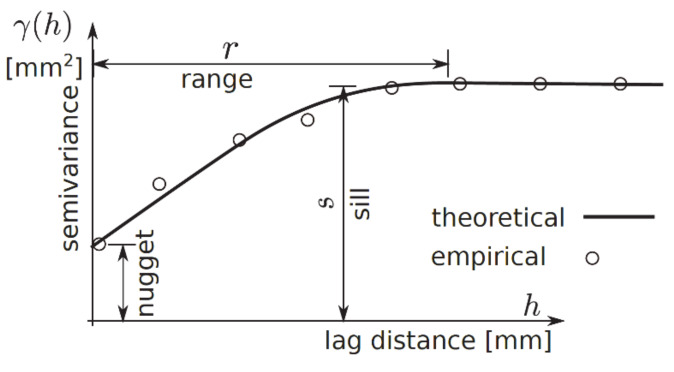
Semivariogam fitting and notation.

Notation and semivariance fittings are presented in Figure 9, where the empirical points and fitted model are presented. When a pair of points appears in an interval, the lag distance between them increases linearly. This concept introduces a statistical measure of “z” variability based on a distance between points which is different from subjective deterministic measures. Moreover, some specific features of semivariograms are commonly used to describe natural observations:

- The distance where the model first flattens is known as the range *r* [L].
- Point or sample locations separated by a distance smaller than the range *r* are spatially autocorrelated, whereas locations farther apart than the range *r* are not.
- The value that the semivariogram model attains at the range *r* is called the sill *s* [L^2^]. The partial sill is the sill minus the nugget [L^2^].
- Theoretically, at zero separation distance (lag = 0 [L]), the semivariogram value is 0 [L^2^]. However, at an infinitesimally small separation distance, the semivariogram often exhibits a nugget [L^2^] effect, which is some value greater than 0. The nugget effect can be attributed to measurement error or spatial sources of variation at distances smaller than the sampling interval, or both. Natural phenomena can vary spatially over a range of scales. Variation at microscales smaller than the sampling distance will appear as part of the nugget effect.

### 2.6. Geostatistical Model Fitting to Empirical Semivariogram—Surface Semivariogram Model (SSM)

Semivariogram modelling governs a step between spatial description and spatial prediction. Geostatistical analysis provides many semivariogram functions to model the empirical semivariogram, as presented in Table 3 and Figure 8 and Figure 9.

The selection of a theoretical model and its fitting procedure is crucial to get a satisfactory prediction of unsampled points. The maximum likelihood or least square regression (including the weighted version) can be used to fit the experimental semivariance. A small and comparable sum of the deviations indicates comparable performance of the models. Therefore, the selection of the semivariogram model is a prerequisite for better performance. It is possible to have several models to choose from. The selection of a satisfactory model requires the balancing of goodness of fit and model complexity. Taken together, a likelihood ratio approach leading to a chi-square test was used in this study.

Semivariograms were prepared with two methods for the CRP normalized surface models:

- for each pair of points from the surface,
- for a combination of points along parallel lines in (0°, 45° or 90°).

**Figure 10:**
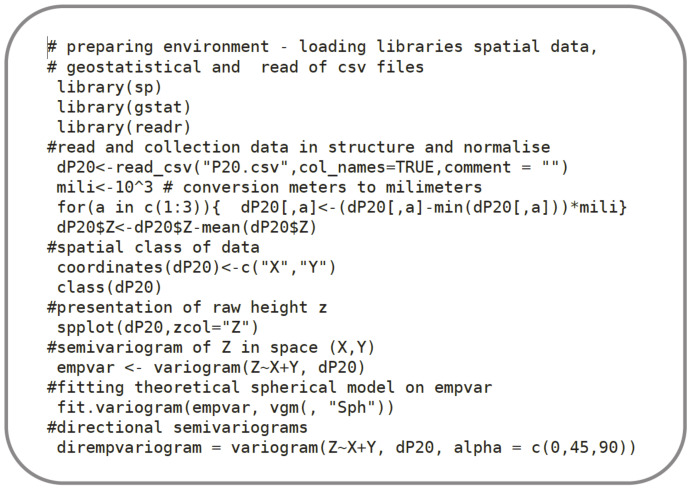
Algorithm No. 1 in R language. Data collection and semivariogram fitting to a sample surface (# starts description line).

At this stage, theoretical semivariograms were fitted to the empirical semivariograms with the use of LSM with uniform weights, as presented in Figure 10.

Algorithm 1 in the R language [22] contains the main part of the fitting code for the spherical model, based on the *Gstat* package [34,35] and SP spatial pack library [36]. The fitted model according to Equation (5) is fully described with two parameters: *r* (range) and *s* (sill), for the zero value of the nugget effect. Both parameters were subsequently used in the process of the generation of a random field described in the next section.

### 2.7. Generation of the Random Field

**Figure 11:**
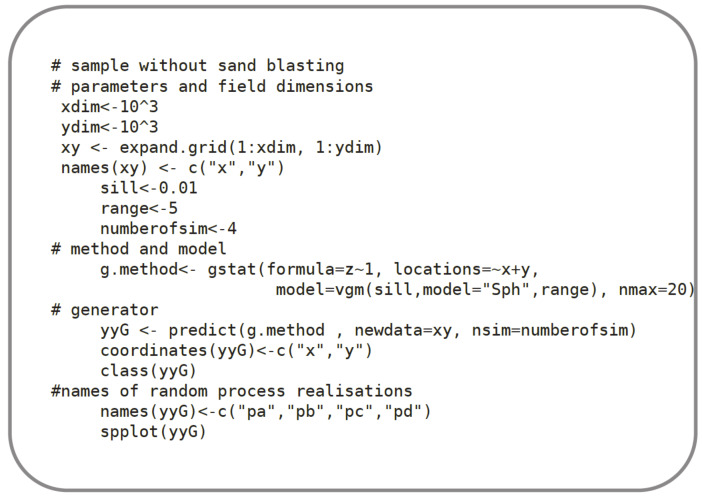
Algorithm No. 2 in R language. Random field generator. (# starts description line).

As mentioned before, semivariograms were used to perform simulations of both the profile and the surface by using the random field theory for the Gaussian process, correlated with the semivariogram. For the field generation, the sequential simulation algorithm was used and coded in the R language [22], as presented in Figure 11. It was assumed that the modelled phenomenon could be described employing the Gaussian random field. The use of the Gaussian random field in Euclidean space assumes that a random process is stationary and isotropic. The symmetrical non-negative correlation function and zero mean value were also assumed. Moreover, the points of the generated samples were evenly distributed on the surfaces and on the profiles.

The random field generator presented in Figure 11 was also based on the *Gstat* package [34,35]. The sequential algorithm operates well on fields of large dimensions (i.e., more than 10^7^ points). To approximate the conditional distribution at a location, this effective method uses only data and simulated values from a local neighborhood. The process is described by parameter *n_max_*. The larger the *n_max,_* the better approximation is with the time consumption increasing exponentially. The selection of the nearest *n_max_* data or previously simulated points is performed in *Gstat* with a bucket quadrate neighbourhood search algorithm [37].

In the sequential Gaussian simulation, a random path through the locations was assumed because with a regular path, the local approximation of conditional distribution (using only the *n_max_* nearest) may have caused spurious correlations. *Gstat* reuses expensive results (neighborhood selection and solution to the kriging equations) for each of the subsequent simulations when multiple realisations of the same field are requested. There is a considerable speed gain in the simulating of multi-fields in a single call compared to several hundred calls, each for simulating a single field. The random number generator used was the native random number generator of the R environment. Besides this, the reproduction of the sampling results (i.e., fixing randomness) was assured by using the random number seed with the *set.seed()* function.

**Figure 12:**
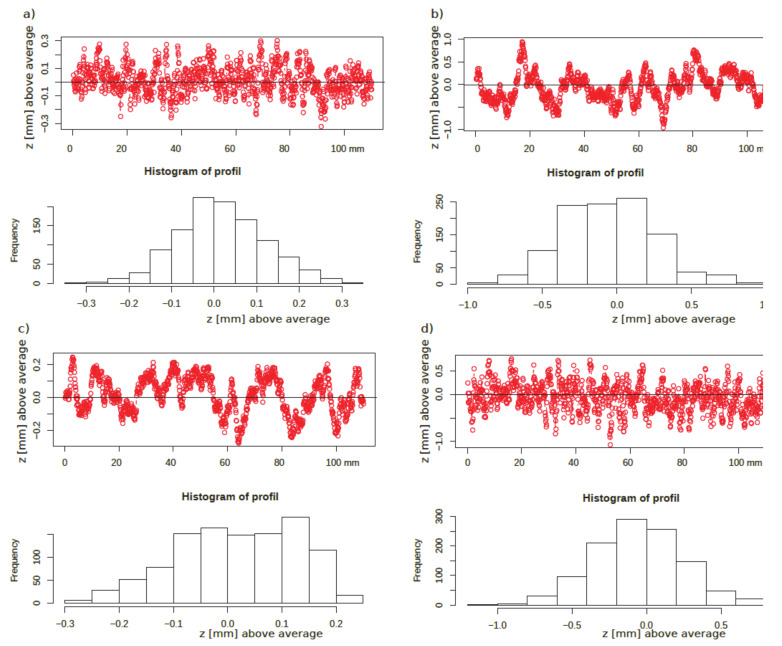
Examples of virtual profiles based on the spherical model with parameters: (**a**) *s* = 0.01 mm^2^, *r* = 1 mm, (**b**) *s* = 0.1 mm^2^, *r* = 5 mm, (**c**) *s* = 0.01 mm^2^, *r* = 5 mm, (**d**) *s* = 0.1 mm^2^, *r* = 1 mm. All profiles are supplied with histograms of *z*. (Horizontal axis - index is a vector of distance in 100 µm).

As a result of the random field generation, a cloud of points with the coordinates (*x,y,z*) was obtained. Examples of the various generated random profiles based on the spherical model, that fits well with relatively even surfaces, are presented in Figure 12.

### 2.8. Surface Roughness Parameters

The random field containing the “*z*” coordinates obtained from the generated virtual surface could be used to calculate any traditional roughness parameters such as average roughness (*R_a_*), mean peak height (*R_pm_*), mean valley depth (*R_vm_*) and many others. However, to explore the available experimental results, [5,10] the mean valley depth (*R_vm_*) was used. *R_vm_* is defined as the average of the maximum valley depth from each sampling length and is given by:

(8) $$R_{vm}=\frac{1}{n}\sum_{i=1}^{n}v_{i}\;,$$

where *v_i_* is the maximum valley depth at each sampling length *i* and *n* is the number of equal sampling lengths.

**Figure 13:**
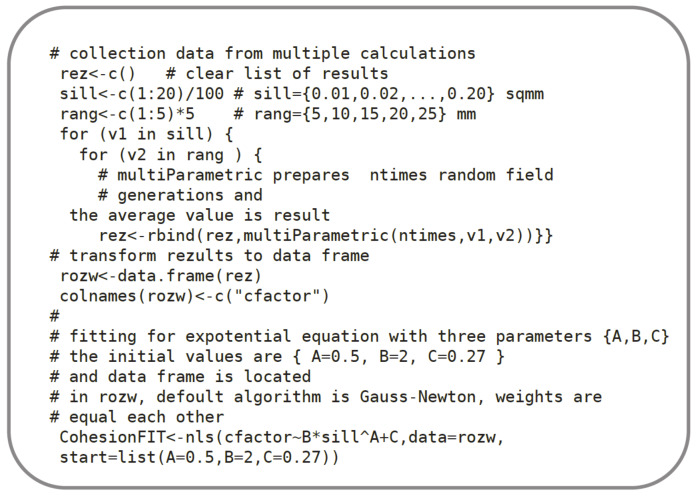
Algorithm No. 3 in R language. Nonlinear least squares (NLS) estimates of the s parameters. NLS use as a default the Gauss–Newton algorithm.

Then, a relationship between the roughness parameter (e.g., *R_vm_*) and semivariogram parameter sill *s* (Figure 9) was obtained with the regression analysis algorithm presented in Figure 13. Evaluation of the roughness parameters is not a necessary step when full test data (i.e. (*x*,*y*,*z*) coordinates) are available because semivariogram parameters can be directly correlated with the strength test results.

### 2.9. Concrete Interface Strength

The final step in the presented method was the transformation of the roughness parameters and, more importantly, the semivariogram parameters into contact bond strength. Factors *c* and *μ* are required (Equation (1)) if [3] is used. According to the results presented in [5,10], relationships between the mean valley depth (*R_vm_*) and the factors are expressed by the following:

(9) $$c=1.062\;R_{vm}{}^{0.145},$$

(10) $$\mu =1.366\;R_{vm}{}^{0.041},$$

However, relationships between the semivariogram parameter *s* (sill) and the *c*, *μ* factors can be achieved with the method presented in Figure 13.

## 3. Results and Discussion

**Figure 14:**
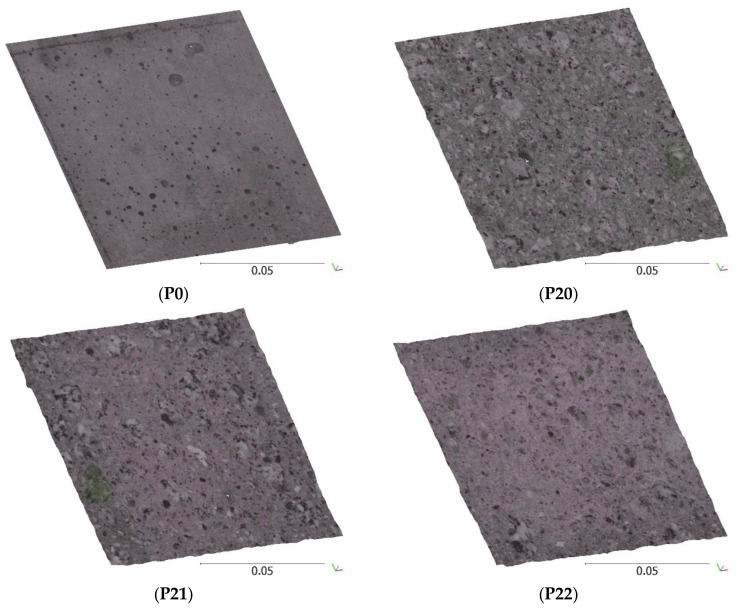

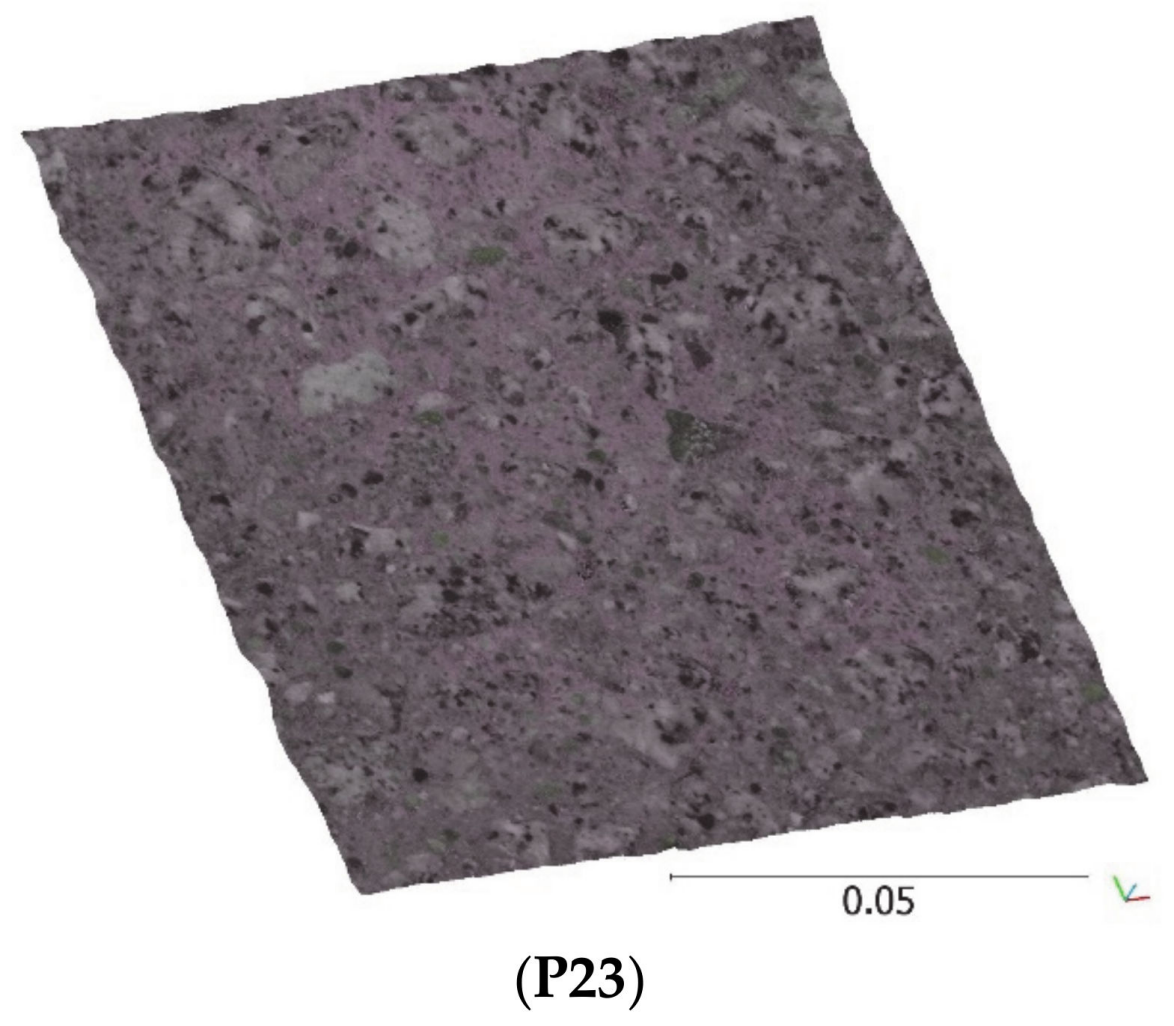
The perspective view of the segments of point clouds obtained from CRP as models of concrete surfaces for samples P0, P20, P21, P22 and P23 (the scale bars are given in meters).

The five concrete surface samples (P0, P20, P21, P22, P23) described in Section 2.2 were examined with the use of CRP. Based on the control points (Figure 7b), the optimization of the camera alignment, as well as the scaling and georeferencing of the models, were performed. The final number of tie points, as well as the obtained model accuracy, are summarized in Table 4. The total error calculated based on six control points did not exceed 0.32 mm and the total error of model scaling calculated based on six scale bars did not exceed 0.16 mm for all five concrete samples. The last step of CRP was to generate a CSV file with the dense point cloud for each concrete sample, as presented in Figure 14. The files contained sets of coordinates and color records (x,y,z,R,G,B). Discrete projection of the surface with a resolution of 100 points per 1 mm (2540 dpi) in the sample plane results in a single file size of approximately 40 MB.

The resulting three-dimensional surface models of the concrete samples were slightly jagged at the edges. For this reason, the concrete sample models were cut symmetrically on each side to a final size of 110 mm × 110 mm and further calculations were performed on the cut samples with the densities presented in Table 4.

**Figure 15:**
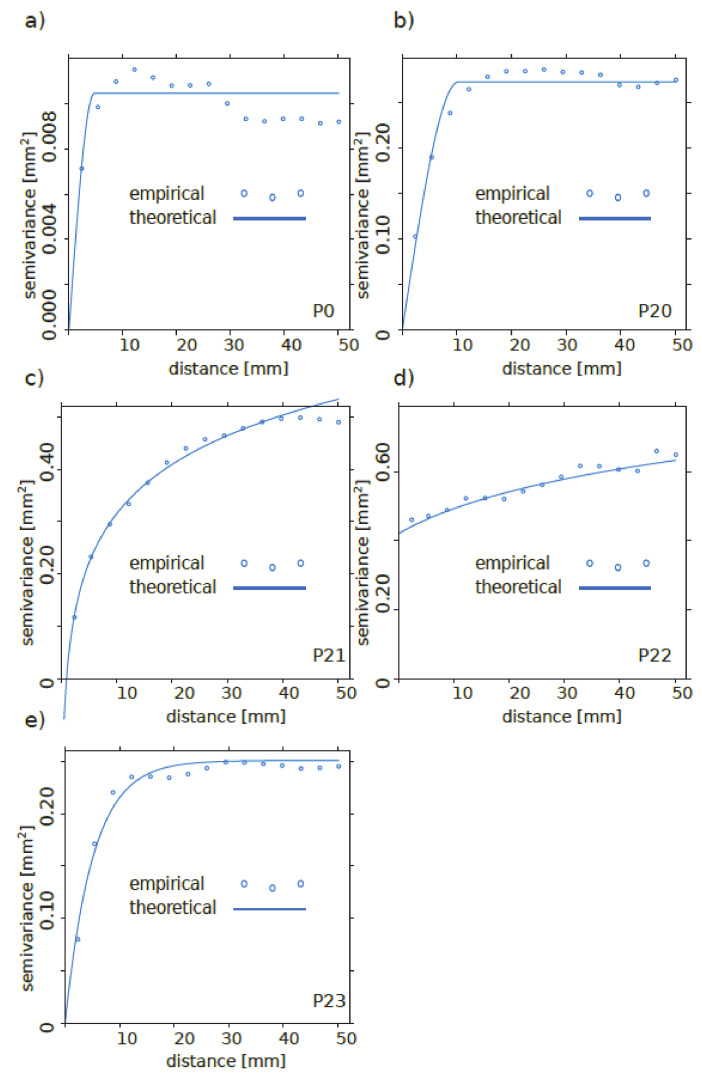
Empirical and theoretical semivariograms for samples: (**a**) P0, (**b**) P20, (**c**) P21, (**d**) P22, (**e**) P23.

For each sample, theoretical semivariograms were fitted to the empirical data with the use of LSM with uniform weights (Algorithm 1 in Figure 10). The “z” coordinates were normalized in reference to the average value and the final semivariogram type was selected based on the shape of the empirical semivariogram, as presented in Figure 15 and Table 5. This selection included the theoretical semivariogram type and method of point selection (each pair from surface or combination along parallel lines).

It is noteworthy that the surface processing type did not change the semivariogram model because the models for samples P0 and P20 were the same and the semivariogram model changed among sandblasted surfaces. This suggests that factors other than the observed parameters can contribute to the semivariogram type.

**Figure 16:**
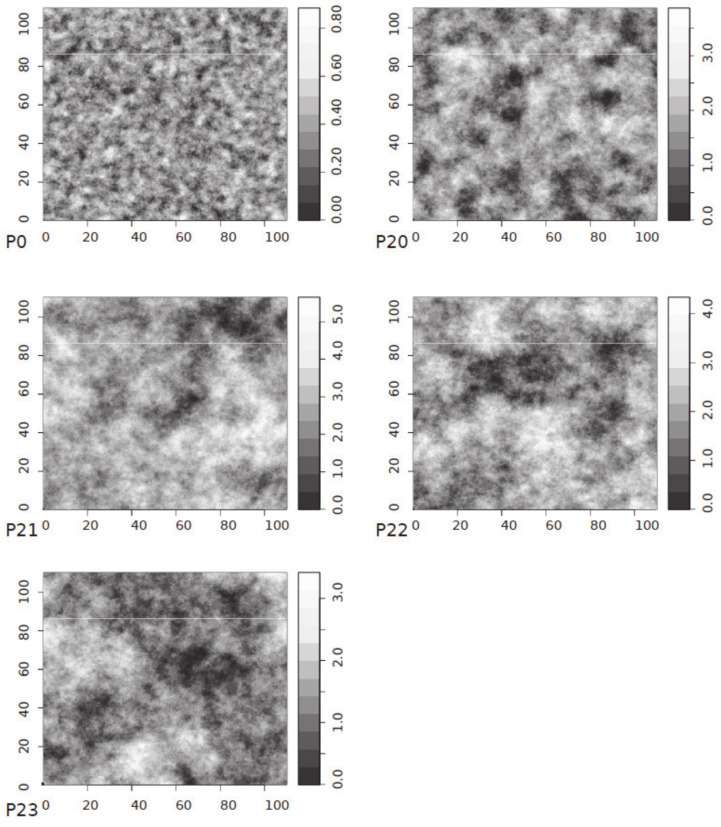
Single random field realisations of the original surfaces P0, P20, P21, P22, P23 (scale values are *z* coordinates in mm).

**Figure 17:**
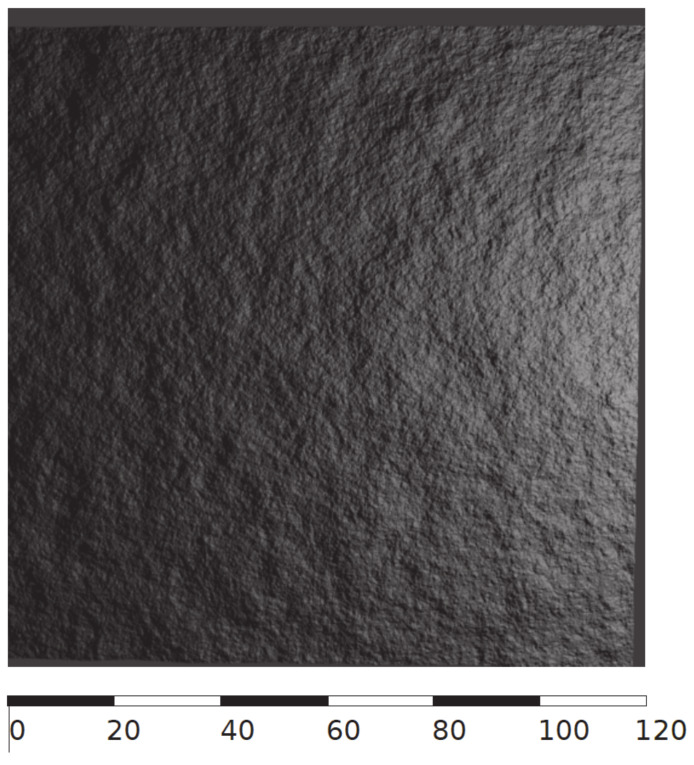
A 3D image of the random field realisation of the original surface P20.

The theoretical semivariograms presented in Table 5 were subsequently used to generate virtual surfaces, as presented in Figure 16 and Figure 17. Furthermore, from these results, the regression models of the mean valley depth *R_vm_*, cohesion factor *c* and friction factor *μ* were approximated as a power function of sill *s*:

(11) $$R_{vm}=B_{1}\cdot s^{A_{1}}+C_{1},\;c=B_{2}\cdot s^{A_{2}}+C_{2},\;\mu =B_{3}\cdot s^{A_{3}}+C_{3},$$

where (A, B, C) is a set of fitting vectors.

**Figure 18:**
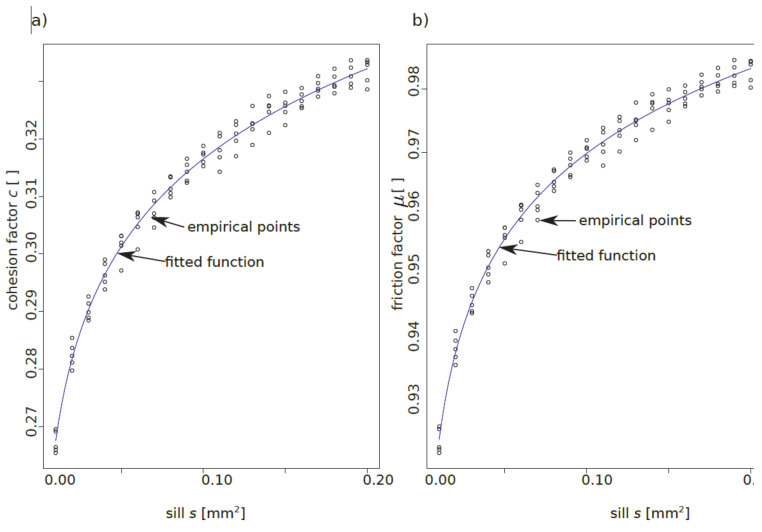
Results of fitting cohesion—*c* (**a**) and friction—*μ* (**b**) factors to the sill—*s* parameter.

In Table 6, the regression parameters can be found, and in Figure 18, the fitting results of factors *c* and *μ* can be found.

The estimation of factors *c* and *μ* as functions of *s* changes Equation (1) to:

(12) $$v_{Rdi}=c\left(s\right)\cdot f_{ctd}+\mu \left(s\right)\cdot \sigma_{n}+\rho \cdot f_{yd}\cdot \left(\mu \left(s\right)\cdot \sin\alpha +\cos\alpha \right)<0.5\;v\;f_{cd},$$

where *v_Rdi_* is the shear strength at the concrete-to-concrete interface; *c(s)* and *μ(s)* are the factors that depend on the interface geostatistical parameter *s* model; ρ is the reinforcement ratio; $\sigma_{n}$ is the external normal stress acting on the interface; *v* is a strength reduction factor; and *f_cd_* is the concrete compressive strength.

If the reinforcement is absent, Equation (12) leads to:

(13) $$v_{Rdi}=c\left(s\right)\cdot f_{ctd}+\mu \left(s\right)\cdot \sigma_{n}$$

or

(14) $$\frac{v_{Rdi}}{f_{ctd}}=c\left(s\right)+\mu \left(s\right)\cdot \frac{\sigma_{n}}{f_{ctd}}.$$

**Figure 19:**
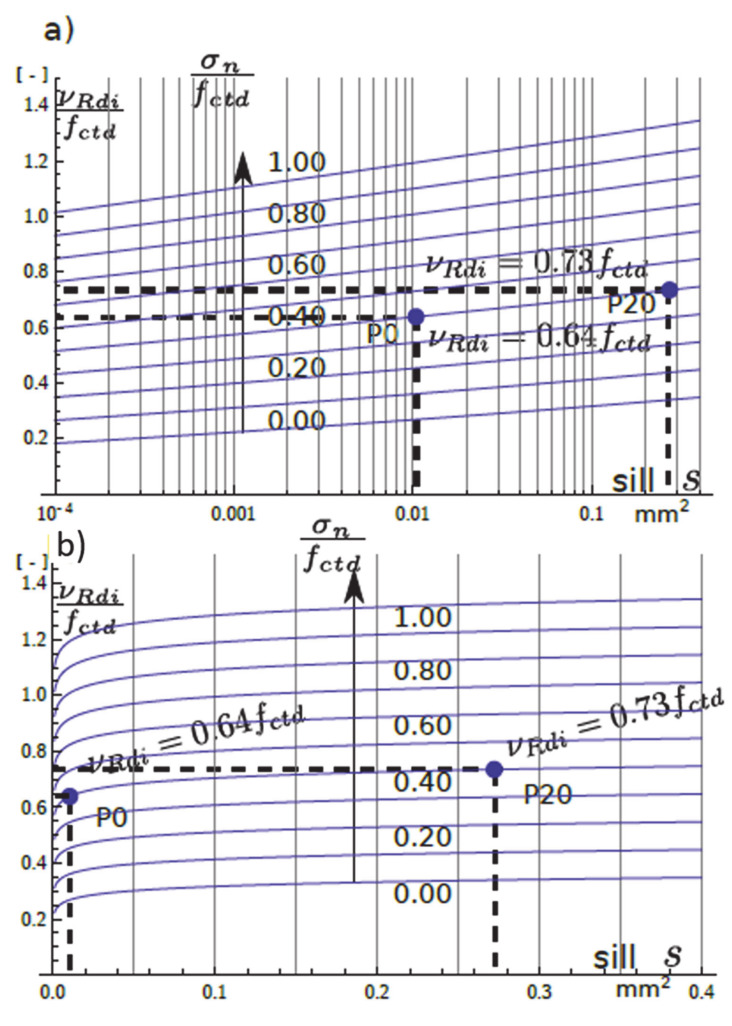
Plots of the relative interface shear strength as a function of the geostatistical sill—s parameter. (**a**) half-logaritmic scale of sill—*s*; (**b**) linear scale of sill—*s*.

Equation (14)’s plots are presented in Figure 19 for the range of normal stress $\sigma_{n}∊\left[0;0.5\right]\;f_{ctd}$. This type of plot could be included in any formal design document.

In the absence of normal stress *σ_n,_* the value of *v_Rdi_*/*f_ctd_* gives the cohesion factor *c* value which, according to [3], varies from 0.025 to 0.4 (and even 0.5 for indented surface). Since the surface of the sample P0 was not processed, it could be regarded as smooth or even very smooth according to [3] *c* = 0.025 − 0.20. The minimum value of *c* in Figure 19 is greater than 0.2 which indicates that the values of *c* in [3] might be too conservative. However, where the factor *μ* is concerned, it does not change very much with surface texture, according to [3] (0.5–0.7 and 0.9 for indented surface). Considering that *σ_n_/f_ctd_* = 1, the plots in Figure 19 give *c* + *μ* values, which for the smooth or very smooth surface is 0.525–0.8. However, in Figure 19 the minimum value of *c* + *μ* = 1.1 which again might be too conservative.

## 4. Conclusions

Instead of traditional roughness parameters, objective statistical parameters were introduced. Thus, a unique random representation of the tested sample was achieved and the randomness of the surface texture was reproduced. Moreover, the sample can be reproduced with random field generation and used if access to the test surface is limited. This feature responds to the need for the non-destructive assessment of strength parameters solely based on concrete surface topography.

Although the developed procedure was tested on a limited number of samples, it exhibited versatile character. The method can be adjusted to the available equipment and field or laboratory conditions. Data collection to create a digital surface model can use any technique (with satisfactory accuracy) and the parameters can be adjusted to the available test data. Although, presented samples did not require much numerical effort, it should be noted that the point cloud size affects the time of numerical calculations.

There exist some limitations of the method applied in this paper, including the need for shadow-free illumination of the sampled element for correct CRP data collection and the use of licensed CRP software. Moreover, despite the simple in situ measurement procedure, the processing time for the model to be obtained can be too long and can affect the time of the construction work. An important feature of this method is a resistance to local texture changes, gaps, aggregate sharp edges, etc., but at the same time these imperfections are relativized and local heterogeneity diminishes because deterministic data are lost. In future research, the influence of surface processing on the semivariogram type should be examined. A full quantitative approach is recommended in the revision of design codes.

## Figures and Tables

**Table 1:** Concrete mix composition.

Cement (C)	Plasticizer	Aggregate		Water (W)	W/C	Bulk Density	Density after 90 d
CEM I 42.5 R	Sikaplast 2545	fine d < 2 mm	coarse 2 mm < d < 16 mm	-	-	-	-
kg/m^3^	kg/m^3^	kg/m^3^	kg/m^3^	kg/m^3^	-	kg/m^3^	kg/m^3^
458.13	5.55	666.37	999.56	187.42	0.41	2317.04	2153.02

**Table 2:** Sample presented in Figure 5—aggregate and cement matrix areas and ratios.

Area	Unit	Concrete Area	Aggregates Area	Cement Matrix Area
A	[mm^2^]	900	433	467
	[%]	100	48.12	51.88
B	[mm^2^]	900	445	455
	[%]	100	49.48	50.52
C	[mm^2^]	900	376	524
	[%]	100	41.81	58.19
D	[mm^2^]	900	416	484
	[%]	100	46.22	53.78
A + B + C + D	[mm^2^]	3600	1671	1929
	[%]	100	46.41	53.59

**Table 3:** Basic semivariogram models.

Model Name	Semivariogram	Equation No.
nugget	$\gamma \left(h\right)=\left\{\begin{array}{c} 0\Rightarrow h=0\\ s\Rightarrow h>0 \end{array}\right.$	(3)
linear with sill	$\gamma \left(h\right)=\left\{\begin{array}{c} \frac{sh}{r}\Rightarrow h\leq r\\ s\Rightarrow h>r \end{array}\right.$	(4)
spherical	$\gamma \left(h\right)=\left\{\begin{array}{c} s\left[1.5\frac{h}{r}-\kappa {\left(\frac{h}{r}\right)}^{3}\right]\Rightarrow h\leq r\\ s\Rightarrow h>r \end{array}\right.$	(5)
exponential	$\gamma \left(h\right)=s\left(1-e^{\frac{-h}{r}}\right)$	(6)
logarithmic	$\gamma \left(h\right)=\left\{\begin{array}{c} 0\Rightarrow h=0\\ s\;\log\left(h+r\right)\Rightarrow h>0 \end{array}\right.$	(7)

*r*—range [L], typically constant value limiting the zone of mutual correlation of points in the model, *s*—sill is the model constant [L^2^], *h*—lag distance, variable in the model function [L], κ—model constant typical as 0.5 [·].

**Table 4:** The number of tie points and assessment of obtained accuracy for the CRP models of the concrete surface samples.

Parameter	Sample Number				
	P0	P20	P21	P22	P23
Number of photos	31	32	26	23	22
Number of tie points	191,205	185,801	184,875	126,506	146,182
Total error of markers in mm	0.26	0.30	0.32	0.27	0.26
Total error of scale bars in mm	0.16	0.16	0.14	0.16	0.15
Original density of point model of concrete sample surface obtained from CRP after cutting to the size of 110 mm × 110 mm (points/mm^2^)/(points/inch^2^)	225/145,161	282/181,935	269/173,548	263/169,677	250/161,290

**Table 5:** Semivariogam fitting results.

Sample Number	Semivariogram Type	Sill mm^2^	Range mm	Method	Surface Type
P0	Spherical	0.0102	4.4744	each pair from surface	not processed, mold touching
P0	Spherical	0.0980	4.513	cop-x ^1^	not processed, mold touching
P0	Spherical	0.0112	4.941	cop-x45 ^2^	not processed, mold touching
P0	Spherical	0.0110	5.012	cop-y ^3^	not processed, mold touching
P20	Spherical	0.2536	10.675	each pair from surface	sandblasting
P20	Spherical	0.2551	11.710	cop-x ^1^	sandblasting
P20	Spherical	0.2510	9.913	cop-x45 ^2^	sandblasting
P20	Spherical	0.2548	12.017	cop-y ^3^	sandblasting
P21	Expotential	0.4832	8.6369	each pair from surface	sandblasting
P21	Expotential	0.4838	8.6367	cop-x ^1^	sandblasting
P21	Expotential	0.4831	8.6353	cop-x45 ^2^	sandblasting
P21	Expotential	0.4827	8.6367	cop-y ^3^	Sandblasting
P22	Logarithmic	0.0791	0.5139	each pair from surface	Sandblasting
P23	Exponential	0.2366	10.4068	each pair from surface	Sandblasting

^1^ cop-x—combination of points along parallel lines, along the x-direction; ^2^ cop-x45—combination of points along parallel lines, along 45 deg to the x-direction; ^3^ cop-y—combination of points along parallel lines, along the y-direction.

**Table 6:** Parameters of regression models *R_vm_*, *c* and *μ*.

Factor/ Parameter	B	A	C	Residual Sum-of- Squares	Number of Iterations to Convergence	Achieved Convergence Tolerance
*R_vm_*	0.752192	0.472575	−0.008818	0.006090	8	5.463 × 10^−8^
*c*	0.58668	0.04188	−0.216210	0.000370	10	2.483 × 10^−6^
*μ*	1.76614	−0.01068	2.78001	0.0003032	6	1.009 × 10^−6^

## References

[1] Cranston W.B., Kamiński M., Wróblewski R. (1996). Aggregate interlock in cracked concrete with excessive crack width. Arch. Civ. Eng..

[2] Birkeland P.W., Birkeland H.W. (1966). Connections in Precast Concrete Construction. ACI J. Proc..

[3] (2004). EN 1992-1-1 Eurocode 2: Design of Concrete Structures -Part 1-1: General Rules and Rules for Buildings.

[4] (2012). Model Code 2010: Final Draft.

[5] Santos P.M.D. Assessment of the Shear Strength between Concrete Layers. Proceedings of the 8th Fib International PhD Symposium in Civil Engineering.

[6] Walraven J.C., Reinhardt H.W. (1981). Theory and Experiments on the Mechanical Behaviour of Cracks in Plain and Reinforced Concrete Subjected to Shear Loading. HERON.

[7] Vecchio F.J., Collins M.P. (1986). Modified Compression-Field Theory for Reinforced Concrete Elements Subjected To Shear. J. Am. Concr. Inst..

[8] Santos P.M.D., Júlio E.N.B.S. (2013). A state-of-the-art review on roughness quantification methods for concrete surfaces. Constr. Build. Mater..

[9] Mohamad M.E., Ibrahim I.S., Abdullah R., Abd Rahman A.B., Kueh A.B.H., Usman J. (2015). Friction and cohesion coefficients of composite concrete-to-concrete bond. Cem. Concr. Compos..

[10] Santos P.M.D., Júlio E.N.B.S. (2014). Interface shear transfer on composite concrete members. ACI Struct. J..

[11] Figueira D., Sousa C., Calçada R., Serra Neves A. (2016). Design recommendations for reinforced concrete interfaces based on statistical and probabilistic methods. Struct. Concr..

[12] (2005). EN 1990. Eurocode–Basis of Structural Design.

[13] Schabowicz K., Ranachowski Z., Jóźwiak-Niedźwiedzka D., Radzik Ł., Kudela S., Dvorak T. (2016). Application of X-ray microtomography to quality assessment of fibre cement boards. Constr. Build. Mater..

[14] Schabowicz K. (2019). Non-destructive testing of materials in civil engineering. Materials.

[15] Hoła J., Sadowski Ł., Reiner J., Stach S. (2015). Usefulness of 3D surface roughness parameters for nondestructive evaluation of pull-off adhesion of concrete layers. Constr. Build. Mater..

[16] Courard L., Piotrowski T., Garbacz A. (2014). Near-to-surface properties affecting bond strength in concrete repair. Cem. Concr. Compos..

[17] Sadowski Ł., Hoła J. (2014). New nondestructive way of identifying the values of pull-off adhesion between concrete layers in floors. J. Civ. Eng. Manag..

[18] Trapko T., Musiał M. (2017). PBO mesh mobilization via different ways of anchoring PBO-FRCM reinforcements. Compos. Part B Eng..

[19] Gaetan C., Guyon X. (2010). Springer Series in Statistics Spatial Statistics and Modeling.

[20] Wackernagel H. (2003). MultivariateGeostatistics. An Itroduction and Applications.

[21] Diggle P.J., Tawn J.A. (1998). Model-based geostatistics. Appl. Stat..

[22] The R Project for Statistical Computing. https://www.R-project.org/.

[23] (2017). EN ISO 14688-2. Geotechnical Investigation and Testing–Identification and Classification of Soil–Part 2: Principles for a Classification.

[24] (2008). EN 933-4. Tests for Geometrical Properties of Aggregates Part 4: Determination of Particle Shapes-Shape Index.

[25] Arias P., Armesto J., Di-Capua D., González-Drigo R., Lorenzo H., Pérez-Gracia V. (2007). Digital photogrammetry, GPR and computational analysis of structural damages in a mediaeval bridge. Eng. Fail. Anal..

[26] Psaltis C., Ioannidis C. An automatic technique for accurate non-contact structural deformation measurements. Proceedings of the International Archives of the Photogrammetry, Remote Sensing and Spatial Information Sciences -ISPRS Archives.

[27] Kwak E., Detchev I., Habib A., El-Badry M., Hughes C. (2013). Precise photogrammetric reconstruction using model-based image fitting for 3D beam deformation monitoring. J. Surv. Eng..

[28] Baqersad J., Poozesh P., Niezrecki C., Avitabile P. (2017). Photogrammetry and optical methods in structural dynamics–A review. Mech. Syst. Signal Process..

[29] Farahani B.V., Barros F., Sousa P.J., Cacciari P.P., Tavares P.J., Futai M.M., Moreira P. (2019). A coupled 3D laser scanning and digital image correlation system for geometry acquisition and deformation monitoring of a railway tunnel. Tunn. Undergr. Sp. Technol..

[30] Muszyński Z., Rybak J., Kaczor P. (2018). Accuracy Assessment of Semi-Automatic Measuring Techniques Applied to Displacement Control in Self-Balanced Pile Capacity Testing Appliance. Sensors.

[31] Agisoft Lens (2016). Version 0.4.2 beta 64bit (build 2399). Lens Calibration Software. Agisoft LLC. http://www.agisoft.com.

[32] (2016). Agisoft PhotoScan Professional Edition. Version 1.2.4 64 bit (build 2399). Multi-View 3D Reconstruction. Agisoft LLC. http://www.agisoft.com.

[33] Isaaks E.H., Srivastava R.M. (1989). An Introduction to Applied Geostatistics.

[34] Pebesma E.J. (2004). Multivariable geostatistics in S: The gstat package. Comput. Geosci..

[35] Pebesma E.J., Wesseling C.G. (1998). Gstat: A program for geostatistical modelling, prediction and simulation. Comput. Geosci..

[36] Bivand R.S., Pebesma E., Gómez-Rubio V. (2013). Applied Spatial Data Analysis with R.

[37] Clarkson K.L. Fast algorithms for the all nearest neighbours problem. Proceedings of the 24th Annual Symposium on Foundations of Computer Science (sfcs 1983).

